# Treatment With High-Hydrostatic Pressure, Activated Film Packaging With Thymol Plus Enterocin AS-48, and Its Combination Modify the Bacterial Communities of Refrigerated Sea Bream (*Sparus aurata*) Fillets

**DOI:** 10.3389/fmicb.2018.00314

**Published:** 2018-02-28

**Authors:** Irene Ortega Blázquez, María J. Grande Burgos, Rubén Pérez-Pulido, Antonio Gálvez, Rosario Lucas

**Affiliations:** Microbiology Division, Department of Health Sciences, University of Jaen, Jaen, Spain

**Keywords:** fish fillets, thymol, bacteriocin, high-hydrostatic pressure, biodiversity

## Abstract

The aim of this study was to determine the impact of activated plastic films with thymol and enterocin AS-48 and high-hydrostatic pressure (HP) treatment on the bacterial load and bacterial diversity of vacuum-packaged sea bream fillets under refrigerated storage for 10 days. The activated film and the HP treatment reduced aerobic mesophiles viable counts by 1.46 and 2.36 log cycles, respectively, while the combined treatment achieved a reduction of 4.13 log cycles. HP and combined treatments resulted in longer delays in bacterial growth. *Proteobacteria* were the dominant phyla in sea bream fillets. The relative abundance of *Firmicutes* increased by the end of storage both in controls and in samples treated by HP singly or in combination with the activated films. The predominant operational taxonomic units (OTUs) found at time 0 in control samples (*Listeria, Acinetobacter, Pseudomonas, Enterobacteriaceae, Chryseobacterium*) rapidly changed during storage (with an increase of *Vibrio, Photobacterium*, and *Shewanella* together with *Cloacibacterium* and *Lactobacillales* by the end of storage). The activated film and the HP treatment induced drastic changes in bacterial diversity right after treatments (with *Comamonadaceae, Methylobacterium, Acidovorax*, and *Sphingomonas* as main OTUs) and also induced further modifications during storage. Bacterial diversity in activated film samples was quite homogeneous during storage (with *Vibrio, Photobacterium*, and *Shewanella* as main OTUs) and approached control samples. HP treatments (singly or in combination with activated films) determined a high relative abundance of *Acinetobacter* (followed by *Pseudomonas* and *Shewanella*) during early storage as well as a higher relative abundance of lactic acid bacteria by the end of storage. The results indicate that the complex dynamics of bacterial populations in the refrigerated sea bream fillets are markedly influenced by treatment and antimicrobials applied.

## Introduction

Fresh fish is a convenient protein ready for preparation of many dishes, but at the same time it is highly perishable. The main cause of deterioration of fresh fish is the metabolic activity of spoilage seafood microorganisms that provoke degradation of organic molecules and fish tissue, loss of essential fatty acids, fat-soluble vitamins and protein functionality, production of biogenic amines, and formation of off-odors (Gram and Dalgaard, 2002).

Natural antimicrobials and non-thermal treatments such as high-hydrostatic pressure (HP), singly or in combination are attractive candidates for preservation of fish and fish products. Previous studies have addressed possible applications of bacteriocins for food preservation, including seafoods (Galvez et al., 2014; Johnson et al., 2017). Enterocin AS-48 is a broad-spectrum circular bacteriocin with a generally-recognized as safe (GRAS) status (Grande Burgos et al., 2014). Immersion in an enterocin AS-48 solution for 1 min delayed bacterial growth and reduced biogenic amine production in sardines during refrigerated storage (Ananou et al., 2014). Spray-application of an enterocin AS-48 solution reduced viable counts of a cocktail of *Listeria monocytogenes* strains on raw hake and salmon fillets as well as on smoked salmon, an effect that was potentiated by bacteriophage P100 (Baños et al., 2016).

Essential oils and their antimicrobial compounds have been studied for preservation of different types of foods (Hyldgaard et al., 2012; Patel, 2015; Pandey et al., 2017). Addition of thymol in combination with other hurdles improved the preservation of fresh packed plaice fillets (Altieri et al., 2005), sea bream, fresh cod, and fresh blue fish burgers (Corbo et al., 2008, 2009; Del Nobile et al., 2009). The antibacterial activity of enterocin AS-48 can be potentiated by essential oils and phenolic compounds (Grande Burgos et al., 2014). Thymol in combination with enterocin AS-48 improved inactivation of *L. monocytogenes* in salads (Cobo Molinos et al., 2009). Combinations of enterocin AS-48 and phenolic compounds such as thymol could also offer new possibilities for fish preservation.

Bacteriocins and essential oils or their antimicrobial compounds have been incorporated on different coating materials with the purpose of preserving fish products, as exemplified by nisin (Neetoo et al., 2008; Lu et al., 2010). However, the only use of natural antimicrobial compounds to inhibit or delay bacterial growth in fish products may require addition of too high concentrations having a negative impact on food flavor. The efficacy of natural antimicrobials incorporated on coating materials could be improved in combination with other hurdles such as HP. HP can be applied on seafood products with several purposes such as inactivation of foodborne pathogens, reduction of biogenic amine production, improving the product shelf life, product texturization, and recovery of fish meat (Murchie et al., 2005; Campus, 2010; Wang et al., 2016). However, pressure treatment of fresh fish flesh formed products whose color deteriorated (cooked appearance) with increasing pressure as well as holding time (Campus, 2010; Wang et al., 2016). Decreasing the intensity of HP treatment reduces the impact on the food properties, but it also decreases its effects on inactivation of microorganisms. Application of HP treatments of low intensity in combination with natural antimicrobials would presumably have a greater effect on inactivation of microorganisms, improving the preservation of fish products.

There is also a growing interest on understanding the complex changes in the food microbiota that may occur during product shelf life as influenced by several factors such as storage temperature, atmosphere, and added antimicrobials. High throughput sequencing (HTS) technology is a powerful tool for studying microbial communities in food systems (Ercolini, 2013; Kergourlay et al., 2015). HTS has proven to be useful in exploring bacterial communities in fish intestine (Ghanbari et al., 2015; Standen et al., 2015; Song et al., 2016) and seafood products (Chaillou et al., 2015; Zhang et al., 2017). In spite of the many studies carried out on the synergistic activities of natural antimicrobials and treatments such as HP, there is still limited information on the effect of such treatments on bacterial populations in fish products. The aim of the present study was to determine the impact of selected treatments (natural antimicrobials thymol and enterocin AS-48 applied on an activated plastic film and HP treatment) on the bacterial load and bacterial diversity of sea bream fillets under refrigerated storage.

## Materials and methods

### Sample preparation

Fresh sea bream (*Sparus aurata*) fillets with skin were purchased from a local supermarket and kept under refrigeration until use (for not longer than 24 h). Fillets were cut into 5 g portions with a sterile knife, and the portions were sealed under vacuum in polyethylene–polyamide bags activated or not with antimicrobials as described below.

Bags (10 × 15 cm) prepared from polyethylene–polyamide film were activated by addition of 1 ml 0.5% thymol (Sigma, Madrid, Spain) plus 1 ml of partially-purified bacteriocin enterocin AS-48 (0.8 mg/ml) prepared as described elsewhere (Abriouel et al., 2003). Briefly, bacteriocin from cultured broths of the producer strain *Enterococcus faecalis* A-48-32 were concentrated by cation exchange chromatography. Bacteriocin concentrates were dialyzed with distilled water for 24 h by using 2,000 molecular weight cut-off benzoylated dialysis tubing (Sigma-Aldrich, Madrid) and filtered through 0.22 μm pore size low protein binding filters (Millex GV; Millipore Corp., Belford, MA, USA) under sterile conditions. The concentrations of antimicrobials used for the study were the ones that achieved highest number of log reductions in viable cell counts while having lowest impact (aromatic odor) on the sensory properties of sea bream fillets among preliminary tests carried out using combinations of 1 ml thymol solutions at 0.25, 0.5, and 0.75% and 1 ml bacteriocin solutions at 0.4 and 0.8 mg/ml. Bags were rubbed by hand to ensure mixing and homogeneous distribution of antimicrobial solutions and incubated for 60 min at ambient temperature to facilitate adsorption of antimicrobials. Then, excess liquid was removed and the bags were allowed to dry for 60 min on filter paper in a biosafety cabinet (Telstar, Madrid, Spain) under UV irradiation. Activated bags were used within 24 h of preparation. Bags not activated with antimicrobials were also kept for 60 min under UV irradiation in a biosafety cabinet.

### High-hydrostatic pressure treatment

The vacuum-packed sea bream fillets were treated by high-HP at low intensity (300 MPa for 5 min at ambient temperature). This treatment was selected among trials at 200, 300, and 400 MPa (5 min each) because it achieved highest number of log reductions while having lowest impact on the natural color of fillets. HP treatment was applied by using a Stansted Fluid Power LTD high pressure Iso-Lab system (model FPG9400B711; SFP, Essex, UK). Come-up speed was 75 MPa/min. Decompression was immediate. Pressurization fluid was water with added 10% propylenglycol. The temperature inside the vessel during treatments ranged between 23 and 27°C. The temperature of samples was 24.5°C at the end of treatment. The samples were placed on ice right after treatment.

The following treatments were applied (two replicates, each one in triplicate): C, controls packed in bags not activated with antimicrobials. AF, samples packed in bags activated with antimicrobials. HP, samples packed in bags not activated with antimicrobials, treated by HP. AF-HP, samples packed in bags activated with antimicrobials, treated by HP. Samples were stored for 10 days at 5°C. At desired incubation times, bags (in triplicate) were removed and the food content was pooled and homogenized with 10 ml sterile saline solution. The resulting homogenate was serially diluted in sterile saline solution and plated in triplicate on trypticase soya agar (TSA; Scharlab, Barcelona, Spain) for determination of total aerobic mesophiles. The average number of colonies obtained after 24–48 h incubation of the plates at 37°C was used to calculate the viable cell concentration [expressed as Log_10_ colony forming units (CFU)/g].

### DNA extraction

For each sampling point, aliquots (5 ml) from food homogenates were mixed in sterile 50 ml test tubes and centrifuged at 600 × *g* for 5 min in order to remove solids. An aliquot (1.5 ml) of the resulting supernatant was then transferred to an Eppendorf test tube and centrifuged at 13,500 × *g* for 5 min to recover microbial cells. The pellet was resuspended in 0.5 ml sterile saline solution each. Then, Propidium Monoazide (PMA™, GenIUL, S.L, Barcelona, Spain) was added to block subsequent PCR amplification of the genetic material from dead cells (Nocker et al., 2007) as described by Elizaquivel et al. (2012). DNA from PMA-treated cells was extracted by using a GenElute™ Bacterial Genomic DNA Kit (Sigma-Aldrich), following instructions provided by the manufacturer. The resulting DNA from the two batch replicates and same sampling point was pooled into a single sample. DNA concentration and quality were measured with a NanoDrop spectrophotometer (Thermo Scientific, United Kingdom).

### DNA sequencing and analysis

The sequence of the V3–V4 region of 16S rRNA gene was used as the taxonomic basis to estimate bacterial populations present in the samples (Caporaso et al., 2011) using Illumina technology essentially as described elsewhere (Grande Burgos et al., 2017). DNA template was adjusted to 10–12 ng in each PCR reaction. The amplicon sequencing protocol targets the V3 and V4 regions of the 16S genes with the primers designed surrounding conserved regions (Klindworth et al., 2013). Following the Illumina amplicon libraries protocol, DNA amplicon libraries were generated using a limited cycle PCR (KAPA HiFi HotStart ReadyMix, KK2602; KAPA Biosystems, Wilmington, MA, USA), then Illumina sequencing adaptors and dual-index barcodes were added to the amplicon. Libraries were then normalized and pooled prior to sequencing. The sample containing indexed amplicons was then loaded onto the MiSeq reagent cartridge v3 (MS-102-3003; San Diego, CA, USA) and onto the instrument along with the flow cell. Automated cluster generation and paired-end sequencing with dual indexes reads was performed (2 × 300 bp run). After demultiplexing, paired end reads were joined together with the fastq-join program (https://expressionanalysis.github.io/ea-utils/). Only reads that had quality value (QV) scores of ≥20 for more than 99% of the sequence were extracted for further analysis. All sequences with ambiguous base calls were discarded. After filtering, sequence reads were assigned to operational taxonomic units (OTUs) based on sequence similarity for each read to 16S rRNA genes from the NCBI nt database by using BLASTN function. Each read was assigned to the taxon corresponding to the Best Blast Hit over a threshold of similarity (e < 1E-15). The sequencing output files were deposited in the Sequence Read Archive (SRA) service of the European Bioinformatics Institute (EBI) database under Accession Number PRJEB22751.

### Statistical analysis

Data on viable cell counts for the different treatments and storage times were analyzed with *t*-test and two-way ANOVA at *P* < 0.05 (Microsoft Excel). Diversity indices Shannon Wiever (H´), Simpson (D) and Chao1 were calculated using the statistical software packages Paleontological Statistics (PAST). Euclidean distance matrix was obtained with SPSS software.

## Results

### Effect of treatments on bacterial inactivation

When control sea bream fillets packed in plastic bags without antimicrobials were stored under refrigeration, viable cell counts (total aerobic mesophiles) increased gradually and were 1.14 log cycles higher by the end of storage period (Table 1). Microbial inactivation was significantly influenced (*P* < 0.05) by activated film packaging, HP treatment, and the combination of activated film and HP. For samples packed in the activated plastic bags, viable cell counts were reduced significantly (*P* < 0.05) by 1.45 log cycles at time 0. However, viable counts obtained during storage were not significantly lower (*P* > 0.05) than untreated controls. The HP treatment applied to samples packed in films without activation with antimicrobials reduced viable cell counts significantly (*P* < 0.05) by 2.36 log cycles. Furthermore, viable counts in the HP-treated samples also remained significantly (*P* < 0.05) lower than controls by 3.21 and 2.62 log cycles for storage times 2 and 5, respectively. However, a remarkable increase in viable counts was noticed by day 7 and after. Application of HP treatment to samples packed in films activated with antimicrobials provided best results, achieving a significant (*P* < 0.05) reduction of 4.13 log cycles. Although viable cell counts for the combined treatment increased during late storage (days 5, 7, and 10), they still remained significantly (*P* < 0.05) lower than the untreated controls for storage times 2, 5, and 7 (by 4.92, 2.67, and 1.63 log cycles, respectively). Compared with the activated film alone, microbial inactivation was influenced significantly (*P* < 0.05) by the combined treatment, which achieved additional and significant (*P* < 0.05) reductions of viable counts (2.67, 4.79, 2.18, and 1.18 log cycles for storage times 0, 2, 5, and 7, respectively). Compared with the single HP treatment, the combined treatment with activated films achieved additional and significant (*P* < 0.05) reductions of viable counts of 1.77 and 1.71 log cycles at storage times 0 and 2, respectively.

**Table 1:** Viable cell counts for total aerobic mesophiles in sea bream fillets stored under refrigeration.

	**Storage time (days)**				
	**0**	**2**	**5**	**7**	**10**
Control	5.97 ± 0.09^a^	6.22 ± 0.15^a^	6.58 ± 0.11^a^	6.55 ± 0.07^a^	7.11 ± 0.15^a^
Activated film (AF)	4.51 ± 0.05^b^	6.09 ± 0.31^a^	6.09 ± 0.31^a^	6.10 ± 0.32^a^	6.93 ± 0.08^a^
HP	3.61 ± 0.19^b^	3.01 ± 0.09^b^	3.96 ± 0.12^b^	5.64 ± 0.38^a^	6.81 ± 0.25^a^
AF-HP	1.84 ± 0.09^c^	1.30 ± 0.25^c^	3.91 ± 0.18^b^	4.92 ± 0.19^b^	7.16 ± 0.36^a^

*Data (Log10 CFU/g) are the average of two replicates ± standard deviation*.

a,b,c *down columns indicate significantly different (P < 0.05) values*.

### Effect of treatments on bacterial diversity

**Figure 1:**
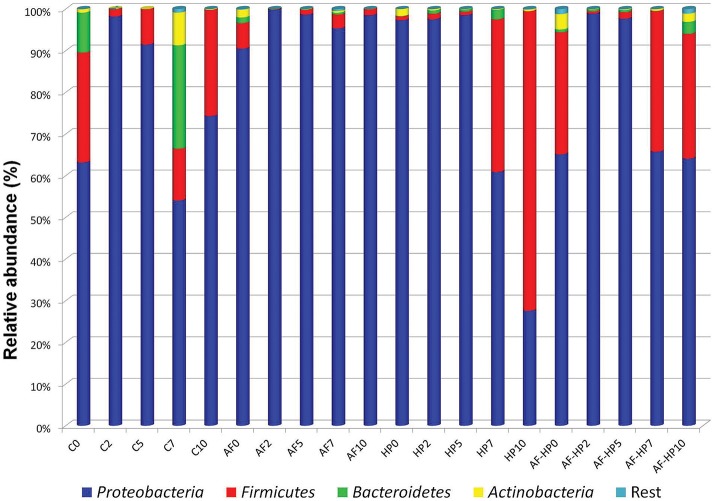
Relative abundance of OTUs based on paired-end 16S rRNA gene sequencing analysis of DNA from refrigerated sea bream fillets. Controls packed in films without activation with antimicrobials (C); samples packed in films activated with thymol plus enterocin AS-48 (AF); samples packed in films without activation and treated by high-hydrostatic pressure (HP); samples packed in activated films and treated by high-hydrostatic pressure (AF-HP). Sampling was performed at days 0, 2, 5, 7, and 10. OTUs were grouped at Phylum level.

The numbers of reads assigned to OTUs was comprised between 77,780 and 190,124 (Table 2). Nevertheless, in spite of the in-depth coverage provided by Illumina sequencing, differences in copy number of the 16S rRNA gene sequencing need to be taken into consideration as well as the variability that may occur between samples. Control samples showed higher alpha diversity values compared with samples packed in the activated films or with samples treated by HP alone (Table 2). In samples from the combined treatments, alpha diversity values decreased at days 2 and 5 but then increased again during storage. *Proteobacteria* were the predominant phylum in the controls as well as in most of the treated samples, with relative abundances ranging from 54.06 up to 99.79% (Figure 1). In control samples, the relative abundance of *Proteobacteria* decreased toward the end of storage, while other phyla (*Firmicutes, Bacteroidetes, Actinobacteria*) increased. For the HP treated samples, the relative abundance of *Firmicutes* increased toward the end of storage, reaching 71.91% at day 10 (HP10). This sample had the lowest relative abundance of *Proteobacteria* (27.58%). For the combined treatments, *Firmicutes* had higher relative abundances both after treatment (AF-HP0, 29.30%) and by the end of storage (AF-HP7, 33.79%; AF-HP10, 29.95%). The rest of phyla (19 in total) had very low relative abundances, and altogether accounted for <1.3% OTUs.

**Table 2:** Number of sequences (reads) and observed diversity for 16S rRNA amplicons for the different samples analyzed in this study.

**Sample**	**N° of reads**	**Shannon index**	**Simpson index**	**Chao 1 index**
**UNTREATED CONTROLS**				
C0	77,780	2.59	0.88	150.6
C2	83,091	1.79	0.77	77.5
C5	190,124	1.91	0.79	116.1
C7	120,747	3.22	0.91	208.2
C10	94,357	2.42	0.86	126
**ACTIVATED FILM**				
AF0	106,044	2.79	0.88	131.1
AF2	92,738	1.64	0.74	113
AF5	83,014	1.92	0.79	108
AF7	84,833	1.98	0.75	142.5
AF10	78,491	1.73	0.74	110.8
**HIGH HYDROSTATIC PRESSURE**				
HP0	99,732	0.99	0.78	107.6
HP2	94,189	1.96	0.71	123
HP5	89,368	1.72	0.73	150.1
HP7	98,761	1.72	0.74	110.5
HP10	81,864	1.94	0.74	132.5
**ACTIVATED FILM PLUS HIGH HYDROSTATIC PRESSURE**				
AF-HP0	114,530	3.34	0.94	172
AF-HP2	90,700	1.68	0.68	132.3
AF-HP5	98,905	1.96	0.76	142.6
AF-HP7	96,927	2.30	0.84	150.8
AF-HP10	97,467	3.44	0.94	157.8

**Figure 2:**
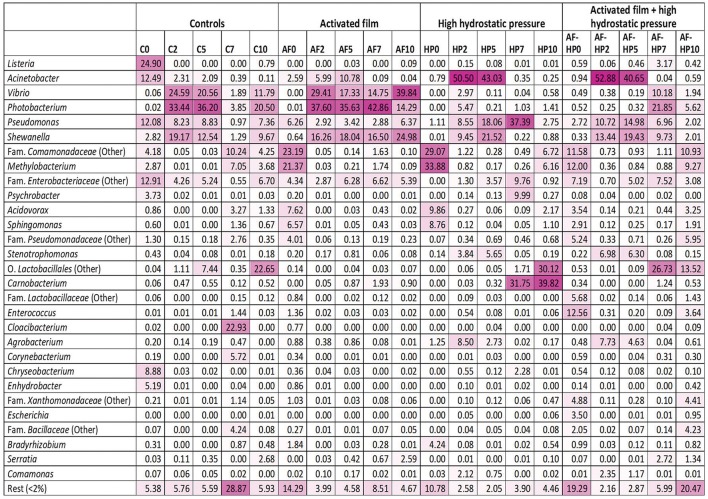
Heatmap of bacterial diversity in sea bream samples based on the relative abundance at genus level. The values represent the relative abundance of OTUs clustered at the Genus level. OTUs not assigned to genus were computed in the nearest higher taxonomic level. Color scale: 0, white; 100, dark magenta. Controls packed in films without activation with antimicrobials (C); samples packed in films activated with thymol plus enterocin AS-48 (AF); samples packed in films without activation and treated by high-hydrostatic pressure (HP); samples packed in activated films and treated by high-hydrostatic pressure (AF-HP). Sampling was performed at days 0, 2, 5, 7, and 10.

Comparison of the relative abundances of OTUs clustered at genus level indicated important differences between controls and treated samples that depended on treatment and also on storage time (Figure 2). For the control samples, OTUs assigned to genus *Listeria* (mainly *Listeria seeligeri*) showed highest relative abundance at time 0 (C0, 24.90%). OTUs assigned to *Acinetobacter, Pseudomonas*, and *Enterobacteriaceae* were also relevant (with relative abundances comprised between 12 and 13%), and to a less extent *Chryseobacterium* (8.88%). The bacterial diversity of control samples changed markedly during storage. By day 2, the main bacterial groups detected at time 0 were displaced by other groups, mainly *Vibrio* (mostly *Vibrio rumoiensis*), *Photobacterium* (mostly *Photobacterium angustum*), and *Shewanella*. The first two groups were still important at the end of storage, but other groups (*Comamonadaceae, Lactobacillales*, and *Cloacibacterium*) also became relevant at some points during mid to late storage.

Activated film packaging induced an early, transient change in the microbiota of samples. The main OTUs detected at time 0 (AF0) belonged to *Comamonadaceae* (23.19%), *Methylobacterium* (21.37%), *Acidovorax* (7.62%), and *Sphingomonas* (6.57%) (Figure 2). However, the relative abundances of these bacterial groups decreased markedly by day 2. Furthermore, the main OTUs detected for the activated film samples during storage (AF2 to AF10) belonged to *Vibrio, Photobacterium*, and *Shewanella*, resembling the bacterial diversity of control samples.

The HP-treated samples (HP0) showed a similar profile as AF0 samples, with *Comamonadaceae* (29.07%), *Methylobacterium* (33.88%), *Acidovorax* (9.86%), and *Sphingomonas* (8.76%) as main OTUs (Figure 2). As in the activated films, these bacterial groups also decreased rapidly in relative abundance during early storage of HP-treated samples. However, *Vibrio* and *Photobacterium* had very low relative abundances during storage. Instead, *Acinetobacter* (mainly *Acinetobacter guillouiae* and *Acinetobacter johnsonii*) showed very high relative abundances at days 2 and 5 (HP2, 50.5%; HP5, 43.03%). *Pseudomonas* and *Shewanella* were also relevant in the HP-treated samples during mid-storage. Furthermore, *Lactobacillales* and *Carnobacterium* became the predominant OTUs during late storage (HP7, HP10).

Samples packed in the activated film and then treated by high-HP (AF-HP0) showed lower relative abundances of *Methylobacterium* (12.00%), *Comamonadaceae* (11.58%), *Acidovorax* (3.54%), and *Sphingomonas* (2.91%) compared to the single HP treatment, and higher relative abundances of *Enterococcus* (12.56%), *Enterobacteriaceae* (7.19%), *Lactobacillaceae* (5.68%), *Xanthomonadaceae* (4.88%), and *Escherichia* (3.50%) (Figure 2). However, the relative abundances of these groups decreased rapidly during storage while other groups resembling the microbiota of the single HP treatment increased. Thus, the predominant OTUs detected in samples from the combined treatment at days 2 and 5 belonged to *Acinetobacter* (AF-HP2, 52.88%; AF-HP5, 40.65%) followed by *Shewanella* and *Pseudomonas* in lower proportions. There was also a change in the microbiota during late storage, with increases in the relative abundances of OTUs assigned to *Photobacterium, Vibrio, Shewanella*, and *Enterobacteriaceae* at day 7, *Comamonadaceae* and *Methylobacterium* at day 10, and *Lactobacillales* at both days 7 and 10.

**Figure 3:**
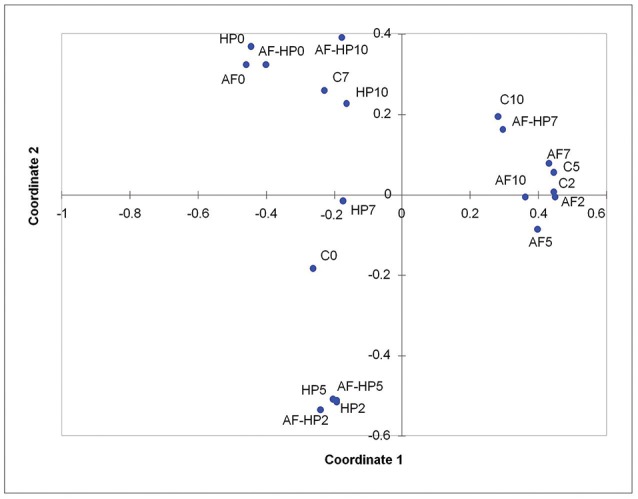
Principal Coordinates analysis (PCoA) of the dissimilarities between controls and treated sea bream samples during refrigerated storage. Controls packed in films without activation with antimicrobials (C); samples packed in films activated with thymol plus enterocin AS-48 (AF); samples packed in films without activation and treated by high-hydrostatic pressure (HP); samples packed in activated films and treated by high-hydrostatic pressure (AF-HP). Sampling was performed at days 0, 2, 5, 7, and 10.

Principal coordinates analysis indicated that most of the activated film samples (AF2 to AF10) as well as control samples C2 and C5 clustered together, as they had high relative abundances for OTUs assigned to *Vibrio, Photobacterium*, and *Shewanella* (Figure 3). Samples just treated by the activated film (AF0), HP (HP0), or both (AF-HP0) formed a separate cluster (with *Comamonadaceae, Methylobacterium, Acidovorax*, and *Sphingomonas* as main OTUs in common). Samples treated by HP and samples from the combined treatment also clustered together for days 2 and 5 (HP2, HP5, AF-HP2, AF-HP5) as they shared in common high relative abundances of *Acinetobacter* followed by *Pseudomonas* and *Shewanella*. The treated samples from late storage HP10 and AF-HP10 also clustered closely, and shared high relative abundances of *Lactobacillales*. Control samples from late storage also had a high relative abundance of *Lactobacillales*, but they clustered more closely to samples from the combined treatment AF-HP7 because they had similar relevant relative abundances of *Vibrio* and *Photobacterium*.

## Discussion

Results presented in this study indicate that reduction of the initial microbial load in sea bream fillets and retardation of its growth during refrigerated storage can be improved considerably by application of combined treatments such as films activated with a mixture of thymol plus the bacteriocin enterocin AS-48 and a mild high-HP treatment. The combined treatment had the advantage of increasing microbial inactivation at the beginning, and still kept viable cell counts in the packed fillets 1.2 log cycles lower than the untreated controls at day 7 of storage. Compared to other methods for bacteriocin addition such as dipping or spraying with bacteriocin solutions, activated films provide the advantage of a slow release of antimicrobials into the medium while at the same time can also provide some protection against bacteriocin degradation by tissue proteases or by complex formation with food components (Gálvez et al., 2007). Although bacteriocins can show synergistic effects with other natural antimicrobials and with non-thermal food processing treatments such as HP (Galvez et al., 2014; Mathur et al., 2017), there are no previous studies on the combined effect of activated packaging with thymol plus enterocin AS-48 and HP treatment. Inability to repair multiple damages caused by the combined action of different antimicrobials most likely results in energy exhaustion and cell death. Furthermore, damage to the outer membrane of Gram-negative bacteria caused by some antimicrobials and/or treatments results in cell permeabilization and faster diffusion of bacteriocins such as enterocin AS-48 to its main cellular target, the bacterial cytoplasmic membrane (Gálvez et al., 1991).

Considering that foods are complex ecosystems containing a mixture of bacterial species that may change in relative abundance during storage depending of different environmental factors such as storage temperature and the presence of antimicrobial substances, among others, it is important to study the changes in the bacterial diversity of foods after application of food preservation methods. The data obtained by HTS can throw light on the behavior of potentially pathogenic and spoilage bacteria in foods during storage. Nevertheless, the influence of the method used to assess the biodiversity on the results needs to be taken into consideration. For example, differences in copy number of the 16S rRNA gene can cause quantification problems. Furthermore, results will also depend on sample variability. Considering these limitations, the results obtained in the present study by HTS revealed that the main bacterial groups detected in the fresh fillets after being packed in vacuum (*Listeria, Acinetobacter, Pseudomonas, Chryseobacterium*, and members of *Enterobacteriaceae*) were rapidly displaced by other bacterial groups during storage (mainly members of *Vibrio, Photobacterium*, and *Shewanella* together with other groups such *Cloacibacterium, Lactobacillales*, and *Comamonadaceae* appearing during late storage). Previous studies reported that *Shewanella putrefaciens* was found to be very important for spoilage of packed cod (Jorgensen et al., 1988) as well as *Photobacterium phosphoreum, Pseudomonas* spp., *Achromobacter* spp., *Acinetobacter* spp., *Flavobacterium* spp., and *Aeromonas* spp. (Dalgaard et al., 1993; Dalgaard, 1995). It is worth mentioning that OTUs for *Listeria* detected in the present study were assigned to *L. seeligeri* and that *L. monocytogenes* was not detected in the sea bream fillets. While isolates of the species *L. seeligeri* are typically hemolytic, this species is generally considered non-pathogenic (Orsi and Wiedmann, 2016). Furthermore, OTUs assigned to *Listeria* had very low relative abundance in all treated samples. *L. monocytogenes* is very sensitive to enterocin AS-48, but it is also known for its high barotolerance and its wide pressure resistance variation between strains (Bruschi et al., 2017). Compared to *L. monocytogenes*, there are scarce data on pressure resistance variations between strains of *L. seeligeri*.

Packing in the activated film induced drastic changes on the sea bream microbiota. All relevant groups in control samples (C0) had reduced relative abundances in samples packed in the activated films (AF0), where OTUs assigned to *Comamonadaceae* and *Methylobacterium* (and to a less extent also *Acidovorax* and *Shewanella*) became predominant. Members of fam. *Comamonadaceae* have been described as part of the culturable microbiota of zebra fish (Cantas et al., 2012), and *Methylobacterium* has been described as a commensal bacterium from the skin of the salmonid brook charr (*Salvelinus fontinalis*) (Boutin et al., 2014). During storage, the bacterial communities of sea bream fillets packed in the activated films seemed to become quite stable, and the main groups detected (*Vibrio, Photobacterium*, and *Shewanella*) were the same as in the untreated controls. These results would be expected since the activated films did not prevent bacterial growth during storage. A possible explanation for the observed low efficacy of activated films during storage could be that added antimicrobials decreased below their minimum inhibitory concentrations upon diffusion from the activated film to the fish tissue, together with perhaps partial degradation of bacteriocin molecules by tissue proteases. The results also suggest that these bacterial groups are less sensitive to the antimicrobials employed, thymol and enterocin AS-48 and/or have a greater capacity to survive in the fish fillets under stress conditions. Since enterocin AS-48 acts on the bacterial cytoplasmic membrane (Gálvez et al., 1991), this bacteriocin has much lower activity against Gram-negative bacteria. Nevertheless, large differences in sensitivity to the bacteriocin have also been reported among Gram-negatives (Gálvez et al., 1989). By contrast, thymol can freely cross the cell wall and therefore inhibit the growth of both Gram-positive and Gram-negative bacteria by interacting with the phospholipid bi-layer of cell membranes, resulting in metabolite dissipation (Hyldgaard et al., 2012). The possible species-dependence of the synergistic action of thymol with enterocin AS-48 deserves to be investigated in further studies.

The HP-treated samples had much lower relative abundances of OTUs assigned to *Vibrio* and *Photobacterium* compared with controls (C2, C5, C10) and with activated film packaging samples (AF2 to AF10). This may suggest a higher sensitivity of these bacterial groups to HP treatment. Compared to the single HP treatment, the combined treatment (activated film-HP) decreased the relative abundance at time 0 of *Comamonadaceae* and *Methylobacterium* and increased that of *Firmicutes* (mainly *Enterococcus* and *Lactobacillaceae*). However, samples from both treatments had similar bacterial compositions during storage. These results could be explained because the activated film alone only had limited effects on bacterial diversity during storage as discussed above. It is also worth mentioning that the main bacterial group detected during early storage of samples treated by HP (singly or in combination with the activated film) was *Acinetobacter*. Genus *Acinetobacter* has been reported as a member of the bacterial community of sea bream (*S. aurata*) larvae early after hatching (Califano et al., 2017) and also as one of the dominant genera of the intestinal microbiota of Antarctic fish (Song et al., 2016). *Acinetobacter* was also predominant in salted bighead carp (*Aristichthys nobilis*) refrigerated fillets (Liu et al., 2017), in farmed sea bream (*S. aurata*) (Parlapani et al., 2013) and in grass carp (*Ctenopharyngodon idellus*) packed in sealed polyvinyl chloride bags during the first 6 days of chill storage (Wang et al., 2014). *Acinetobacter* was also detected in common carp (*Cyprinus carpio*) fillets packed in air or under vacuum and stored under refrigeration (Zhang et al., 2015). In the present study, most OTUs included in this group were assigned to the environmental species *A. guillouiae* and to *A. johnsonii*, which rarely causes human infections (Montaña et al., 2016). Nevertheless, further studies need to be carried out in order to determine the potential risks of *Acinetobacter* in the sea bream fillets as well as resistance of the bacterium to inactivation by HP. *Acinetobacter*, together with *Pseudomonas* and *Shewanella* seemed to be the main spoilage bacteria during early storage in the HP-treated samples and also in the samples from the combined treatment (AF-HP). The observed higher relative abundances of these bacterial groups could be explained by several factors such as higher capacity to repair cellular damage induced by HP treatment, a higher capacity for utilization of available nutrients, ammensalism, or inactivation of competitors by HP treatment.

*Carnobacterium* and *Lactobacillales* were detected at high relative abundances in the HP-treated samples during late storage. *Lactobacillales* were also the most relevant group during late storage of samples from the combined treatment (AF-HP7, AF-HP10). These results would suggest that HP had selected lactic acid bacteria. Similar results have been reported by high-throughput sequencing studies in mango pulp (Pérez Pulido et al., 2017) and by culture-dependent methods in meat products treated by HP (Garriga et al., 2004; Diez et al., 2009; Han et al., 2011).

Altogether, results from the present study reveal the complexity of bacterial populations in sea bream fillets and how these can be influenced by application of different preservation methods and storage time. The activated film and the HP treatment distinctively selected for different bacterial communities during food storage. While the HP treatment (applied singly or in combination) was most clearly associated with a predominance of OTUs assigned to lactic acid bacteria toward the end of storage, the high relative abundance of OTUs assigned to *Acinetobacter* in the HP-treated samples deserves further investigation.

## Author contributions

IO carried out sample preparation, microbiological analysis and DNA extraction for biodiversity studies. MG participated in supervision of the experimental work and interpretation of data. RP-P contributed with high-HP processing, data analysis and preparation of graphical material for the manuscript. RL was responsible for planning and supervision of the study. RL and AG carried out global analysis of the results and wrote the manuscript.

### Conflict of interest statement

The authors declare that the research was conducted in the absence of any commercial or financial relationships that could be construed as a potential conflict of interest.
